# Institutional Amenities

**Published:** 1898-10-29

**Authors:** 


### The Institutional Workshop.

### INSTITUTIONAL AMENITIES.

By a Member of the London Press.

II.

It would be quite possible to go cn adding to the list of amenities as practised in some of our charitable institutions, some of which were described in a previous article. But I am not a grievance monger, and I intensely dislike dealing with the kind of criticism that I am now engaged in. At the same time, I think this question of institutional amenities needs to be discussed before those who are most concerned. And I would like the stigma of a want of courtesy—which undoubtedly at the present time rests on these—removed from our institutions. I very commonly hear both Press men and Press women saying in newspaper offices, "I don't mind what 'assignment' the editor gives me so long as it doesn't include a hospital. Hospital people don't know the value of time, and consequently they keep one waiting about half the day before answering a simple question; and then they don't always answer particularly courteously." Hearing such criticism so constantly applied in newspaper offices to hospital officials impelled me to write as I have done. My interest in hospitals is a very special one, and I think few newspaper writers are better acquainted than I am with the internal workings of our home institutions, as well as those of in many foreign countries. And this fact, while it allows me to make more allowances, renders me more regretful that some institutions which do such admirable work should present a discourteous front to the public. If I were to bombard a hospital, my note-book in my hand, demanding information as to an alleged "abuse" or reported neglect to a patient, I should not expect to be effusively welcomed. At the same time, I contend, even if one had to perform so unpleasant a mission as that suggested, such an inquirer should be met with every civility and courtesy. But when I approach a hospital in friendly spirit, anxious to put any literary eloquence that may be mine at the disposal of such an institution, I most certainly expect to receive the average amenities of civilised life. More than this one neither asks nor desires.

A spirit of courtesy or the reverse soon permeates an institution. If the matron or secretary are somewhat curt and short in their manner to strangers, the porter and lift-boy will take it on themselves to be brusque and familiar, and the spirit of discourtesy will spread as though it were an infectious fever. I have invariably noticed that in those hospitals fortunate enough to possess a staff of sisters who are pleasant in manner and treat the casual visitor with that consideration one gives to the stranger under one's own roof—and the ward is for the time being the sister's home—that the nurses also take the same trouble." It has frequently fallen to my lot to be directed to a ward at which I have duly knocked, waited, knocked again, waited, and entered. The staff nurse, seated at the further end of the ward, looks up, and fixing the stranger with her eyes allows him to walk the entire length of the ward towards the Royal chair, where he is expected humbly to explain his presence and his mission. I never in the least resent this crushing procedure. The nurse may or may not be a gentlewoman, and her conduct on such an occasion at once decides the problem. The nurse, however, is not a permanent or an authoritative official, and her manner to the stranger does not involve the honour and well-being of the institution as is the case when the higher officials fail. At the same time it must be acknowledged that any want of courtesy shown in a hospital—even by the youngest probationer—leaves an unpleasant impression on the mind of a visitor. There cannot but be a suspicion in the background that if the well-dressed visitor is treated thus, the shabby and poor out-patient and the ill-clad friends of the sick will meet with a more accentuated and active want of consideration. And this is a by no means popular or pleasant suspicion for any public institution to impress on the consciousness of the stray visitor. I have visited the leading hospitals and institutions of the United States, and, though the tone of American manners is not generally held to compass the "good form" of Great Britain, I cannot recall an instance of any want of courtesy, either from a door-keeper, an elevator-boy, or a permanent official. To class Chicago and courtesy together would be a union of ideas which would evoke a broad smile from the average Britisher. But from an extended acquaintance with the institutions of Chicago, seen from the point of view of an outsider, I would guarantee that any well-behaved, decent-looking person who made a tour of the Chicago institutions would receive every consideration and courtesy. Neither would he need a letter of introduction to ensure pleasant treatment. A letter of introduction seems almost as though it were designed, like a coat of mail, to ward off a hostile attack. It should not be necessary, at any rate in philanthropic institutions, in order to protect the stranger from the want of courtesy of other strangers. Chicago was specially mentioned—not by any means because it is superior in point of manners to New York and the Eastern States generally, but because Chicago has become associated in the popular mind with an extreme of brusquerie—probably in the minds of those who have not been there.

Going on to Canada, the same formality and red-tapeism confronts one that is met with in London. One often experiences in Canadian hospitals the very faults I have had to criticise in my remarks as to the amenities of London institutions. It is, on the other hand, a rare pleasure to visit a French or a German hospital. There may be many points of criticism as to methods and management. At the present time I am dealing with manners. And the manners of France and those made in German hospitals are up to a very high standard. I have also visited Italian hospitals. It would be difficult to forget how dirty some of them were; it would be impossible to forget the courtesy of all their officials.

In Norway, too, I have had most delightful experiences of institutional amenities, as also in Portugal and other parts of the world, that it is not necessary for the purposes of my articles to particularise or describe. But the fact remains that most other nations can give us points as to our attitude toward the stranger. This is not an individual discovery—the comparison has frequently been made. My object is to localise the argument to fit our institutions, and to point the moral that our hospitals should be centres of civilisation, where the stranger—from the ragged frequenter of the out-patient department up to the rich visitor with powers of bequeathment and valuable legacies—should be accorded a kindly welcome and polite consideration. The whole matter resolves itself into a question of ethics, and it would be as simple to impress the principle that courtesy is part of the hospital routine as it is to establish the principle that the entrance must be swept or that surgical instruments must be rendered aseptic. The code of etiquette as to the reception of the senior medical staff is, as everybody knows, stringently adhered to. It is only a step further to inculcate ideas of hospitality towards the stranger. It seems a pity to allow good manners to lie hid behind a bushel. For these are such charming commodities that they should be brought out and used constantly. And courtesy is among the few goods of this transient life which does not spoil by everyday use, and which costs nothing to give or receive.

